# T168. STRUCTURAL COVARIANCE AND CORTICAL REORGANIZATION IN SCHIZOPHRENIA: AN MRI-BASED MORPHOMETRIC STUDY

**DOI:** 10.1093/schbul/sby016.444

**Published:** 2018-04-01

**Authors:** Lena Palaniyappan, Peter Liddle

**Affiliations:** 1 University of Western Ontario; 2 University of Nottingham

## Abstract

**Background:**

In patients with schizophrenia, distributed abnormalities are observed in grey matter volume. A recent hypothesis posits that these distributed changes are indicative of a plastic reorganization process occurring in response to a functional defect in neuronal information transmission. We investigated the structural covariance across various brain regions in early-stage schizophrenia to determine if indeed the observed patterns of volumetric loss conform to a coordinated pattern of structural reorganization.

**Methods:**

Structural MRI scans were obtained from 40 healthy adults and 41 age, gender and parental socioeconomic status matched patients with schizophrenia. Volumes of grey matter tissue was estimated at regional level across 90 atlas-based parcellations. Group level structural covariance was studied using a graph theoretical framework.

**Results:**

Patients had distributed reduction in grey matter volume, with high degree of localized covariance (clustering) compared to controls. Patients with schizophrenia had reduced centrality of anterior cingulate and insula but increased centrality of the fusiform cortex, compared to controls. Simulating targeted removal of highly central nodes resulted in significant loss of the overall covariance patterns in patients compared to controls.

**Discussion:**

Regional volumetric deficits in schizophrenia are not a result of random, mutually independent processes. Our observations support the occurrence of a spatially interconnected reorganization with systematic de-escalation of conventional ‘hub’ regions. The resulting morphological architecture may be primed for compensatory functions, albeit with a high risk of inefficiency.

